# Copper metabolism of astrocytes

**DOI:** 10.1186/2193-1801-4-S1-L3

**Published:** 2015-06-12

**Authors:** Ralf Dringen, Ivo Scheiber, Felix Bulcke

**Affiliations:** Centre for Biomolecular Interactions Bremen, Faculty 2 (Biology/Chemistry), University of Bremen, Bremen, Germany

**Keywords:** Astrocytes, metals, oxidative stress

Copper is an essential trace element which is involved in many important cellular functions [1]. However, excess of copper can impair cellular functions by copper-induced oxidative stress. In brain, astrocytes are considered to play a prominent role in antioxidative defence as well as in the copper homeostasis [1, 2]. To investigate uptake, toxicity, storage and export of copper in astrocytes, we used primary rat astrocyte cultures as model system. Cultured astrocytes efficiently take up copper ions predominantly by the copper transporter Ctr1 and the divalent metal transporter DMT1. In addition, copper oxide nanoparticles are rapidly accumulated by astrocytes, most likely by endocytotic processes. Astrocytes tolerate moderate increases in intracellular copper contents very well. However, if the specific cellular copper content exceeds after exposure to copper or copper oxide nanoparticles a threshold level of around 10 nmol copper/mg protein, accelerated production of reactive oxygen species and compromised cell viability were observed. Upon exposure to sub-toxic concentrations of copper ions or copper oxide nanoparticles, astrocytes increase their copper storage capacity by upregulating the cellular contents of glutathione and metallothioneins. In addition, cultured astrocytes have the capacity to export copper ions which is likely to involve the copper-transporting ATPase 7A. The ability of astrocytes to efficiently accumulate, store and export copper ions suggests that astrocytes play a key role in brain copper homeostasis and that an impairment of astrocytic functions may be involved in diseases which are connected with disturbances in brain copper metabolism.
